# Association between Metabolite Profiles, Metabolic Syndrome and Obesity Status

**DOI:** 10.3390/nu8060324

**Published:** 2016-05-27

**Authors:** Bénédicte Allam-Ndoul, Frédéric Guénard, Véronique Garneau, Hubert Cormier, Olivier Barbier, Louis Pérusse, Marie-Claude Vohl

**Affiliations:** 1 Institute of Nutrition and Functional Foods (INAF), Laval University, Quebec City, QC G1V0A6, Canada; allamndoulbenedicte@gmail.com (B.A.-N.); Frederic.Guenard@fsaa.ulaval.ca (F.G.); Veronique.Garneau@fsaa.ulaval.ca (V.G.); cormier.hubert@yahoo.com (H.C.); louis.perusse@kin.ulaval.ca (L.P.); 2 School of Nutrition, Laval University, Quebec City, QC G1V0A6, Canada; 3 Laboratory of Molecular Pharmacology, CHU-Quebec Research Center, and Faculty of Pharmacy, Laval University, Quebec City, QC G1V4G2, Canada; Olivier.barbier@crchul.ulaval.ca; 4 Department of Kinesiology, Laval University, Quebec City, QC G1V0A6, Canada

**Keywords:** metabolic syndrome, obesity, metabolites

## Abstract

Underlying mechanisms associated with the development of abnormal metabolic phenotypes among obese individuals are not yet clear. Our aim is to investigate differences in plasma metabolomics profiles between normal weight (NW) and overweight/obese (Ov/Ob) individuals, with or without metabolic syndrome (MetS). Mass spectrometry-based metabolite profiling was used to compare metabolite levels between each group. Three main principal components factors explaining a maximum of variance were retained. Factor 1’s (long chain glycerophospholipids) metabolite profile score was higher among Ov/Ob with MetS than among Ov/Ob and NW participants without MetS. This factor was positively correlated to plasma total cholesterol (total-C) and triglyceride levels in the three groups, to high density lipoprotein -cholesterol (HDL-C) among participants without MetS. Factor 2 (amino acids and short to long chain acylcarnitine) was positively correlated to HDL-C and negatively correlated with insulin levels among NW participants. Factor 3’s (medium chain acylcarnitines) metabolite profile scores were higher among NW participants than among Ov/Ob with or without MetS. Factor 3 was negatively associated with glucose levels among the Ov/Ob with MetS. Factor 1 seems to be associated with a deteriorated metabolic profile that corresponds to obesity, whereas Factors 2 and 3 seem to be rather associated with a healthy metabolic profile.

## 1. Introduction

Metabolic syndrome (MetS) is defined as the association of obesity, insulin resistance (IR), hypertension and dyslipidemia [1]. MetS predisposes an individual to several metabolic diseases such as type 2 diabetes and cardiovascular diseases (CVD) [2]. Obesity is mainly considered to be responsible for the rising prevalence of MetS [2], associated with higher plasma triglyceride (TG) levels, lower high density lipoprotein-cholesterol (HDL-C) levels, hyperglycemia and increased CVD risk [1]. The prevalence of obesity has been increasing dramatically worldwide over the past three decades [3].

Obesity results from a complex interaction between predisposing genetic factors and changes in environmental factors such as diet. It is recommended that obesity be the primary target of intervention for MetS [4]. Nevertheless, it is important to point out the fact that 10%–30% of obese individuals are insulin sensitive and have normal plasma lipid profile and blood pressure, thus being considered as obese but metabolically healthy [5,6,7].

Metabolites are intermediate products of different metabolic pathways. Their levels can be modulated by genetic factors, environmental factors or gene–environment interactions. In recent years, advanced laboratory techniques, such as metabolomics have been developed. It has allowed the investigation of a large number of metabolites in human biological fluids or tissues [8]. Metabolomics is a powerful tool which provides the possibility to study metabolite differences between several groups at any given time and thus allows capturing the dynamic physiological conditions corresponding to disease outcomes or metabolic alterations.

Several studies used metabolomics to investigate metabolite profiles associated with obesity. For instance, Lee *et al*. observed a shift in metabolite composition in obese individuals compared to normal-weight individuals [9]. In a study examining the influence of fat free mass on metabolite profile, Jourdan *et al*. showed that the serum metabolite composition was strongly associated with obesity stages [10]. Therefore, investigation of serum metabolite concentrations provides an ideal way to uncover the underlying mechanisms associated with the development of abnormal metabolic status in obese individuals. We hypothesize that metabolite profiles differ between lean and obese subjects with or without MetS. The aim of the present study was to apply comprehensive metabolic profiling tools to gain broader understanding of metabolic differences between lean and obese subjects with or without MetS.

## 2. Materials and Methods

### 2.1. Subjects

Two hundred subjects aged between 18 and 55 years were randomly selected from the INFOGENE study [11]. The recruitment took place between May 2004 and March 2007 in the Quebec City metropolitan area. The final study sample consisted of 664 individuals. Participants were recruited using advertisements in local newspapers and radio stations, in addition to electronic group messages sent to university and hospital employees. During their visit at the clinical investigation unit, participants completed a questionnaire on socio-demographic characteristics and lifestyle habits. All participants signed a written consent to participate in this study that has been approved by the ethics committee of Laval University.

### 2.2. Anthropometric Measurements

A trained research assistant measured height, weight, waist (WC) and hip circumferences. Systolic (SBP) and diastolic (DBP) blood pressure were measured after a 5 min rest while participants were lying on their backs, with arms and legs uncrossed. Fat and lean masses were assessed using a bioelectrical impedance meter (101-RJL Systems, Detroit, MI, USA). Body mass index (BMI) was calculated by dividing weight in kilograms by height in meters squared. The obesity status of the participants was assessed using BMI and the body fat mass percentage. Subjects were classified in the overweight and obese (Ov/Ob) group if they had a BMI ≥ 25 kg/m^2^. Body fat ≥25% of total body fat mass in men and ≥30% in women were considered to classify an individual as being obese [12].

### 2.3. Biochemical Parameters

Fasting blood samples were obtained from an antecubital vein into vacutainer tubes containing Ethylenediaminetetraacetic acid (EDTA) after a 12 h overnight fast. Total cholesterol (total-C) and TG levels were determined from plasma and lipoprotein fractions using the Olympus AU400e system (Plympus America Inc., Melville, NY, USA). A precipitation of low-density lipoprotein cholesterol (LDL-C) fraction in the infranatant with heparin-manganese chloride [13] was used to obtain the HDL-C fraction. LDL-C concentrations were estimated using the Friedewald’s equation [14,15]. Radioimmunoassay with polyethylene glycol separation was used to measure fasting insulin [16]. Fasting glucose concentrations were enzymatically measured [17]. Homeostasis model assessment of insulin resistance (HOMA-IR) was obtained using: (fasting glucose × fasting insulin)/22.5 [18].

### 2.4. Metabolite Profiling

To assess the metabolite profiling, the Absolute ID p180 Kit (Biocrates Life Sciences AG, Innsbruck, Australia) for mass spectrometry was used. The quantification of 95 metabolites was done for 200 participants. Metabolites were composed of 67 Glycerophospholipids (GPs), 12 Acylcarnitines (ACs), 10 Sphingolipids (SGs) and 6 amino acids (AA). For GPs, ACs and SGs x:y notation was used, x denotes the number of carbons in the side chain and y the number of double bonds. The lipids were subdivided into different classes: 28 Phosphatidylcholines diacyl (PCaa); 36 Phosphatidylcholines acyl-alkyl (PCae); 3 LysoPhosphatidylcholines acyl (LysoPC); 4 Hydroxyshingomyelins; and 6 Shingomyelins (SM). GPs are differentiated with respect to the presence of ester (a) and ether (e) bonds in the glycerol moiety. Two letters indicate that the first and second positions of glycerol unit are bound to a fatty acid residue, while a single letter (a or e) indicates a bond with only one fatty acid residue. The concentration of all metabolites was reported in μM. Metabolites for which more than half of the values were below the limit of detection and or with standard out of range were excluded.

### 2.5. Statistical Analyses

Transformations were applied to the variables that were not normally distributed. Logarithmic transformations were performed for fasting insulin, total-C and HDL-C, and an inverse transformation for TG and fasting glucose. All analyses were conducted on age- and sex-adjusted data; analysis of variance was used to underline the differences in metabolic characteristics between obese/overweight with or without MetS and normal-weight participants with or without MetS. Individuals with MetS had at least 3 of the following five criteria: waist circumference (WC) >88 cm for women and 102 cm for men, fasting plasma triglycerides (TG) ≥1.7 mmol/L, high-density lipoprotein cholesterol (HDL-C) levels ≤1.03 mmol/L for men and 1.29 mmol/L for women, glucose levels ≥ 5.6 mmol/L and resting blood pressure ≥130/85 mmHg.

The significance level of all analyses was set at *p* ≤ 0.05. Differences between groups were assessed using the least square means. Principal component analysis (PCA) was used to reduce the large number of correlated metabolites into clusters of fewer uncorrelated factors. Factors derived from PCA that had eigenvalues greater than or equal to 1 were identified and considered for further analyses. Metabolites with a factor loading ≥0.50 or −0.50 were reported as composing a given PCA-derived factor. Then, scoring coefficients were used to calculate baseline metabolite factor scores for each individual. These scores corresponded to the sum of metabolic signature groups multiplied by their respective factor loading. These scores represented the degree of each participant’s metabolic signature conforming to each factor. To detect association between factors and MetS criteria, Pearson correlations were used. All statistical analyses were performed using SAS statistical software version 9.2 (SAS Institute, Inc., Cary, NC, USA).

## 3. Results

### 3.1. Descriptive Characteristics

Descriptive characteristics of study participants are presented in Table 1. Groups were defined according to BMI and MetS status. Ov/Ob individuals with or without MetS were older than normal weight (NW) subjects. Among obese individuals, subjects with MetS were older than those without MetS. As expected, WC and fat mass increased progressively with the deterioration of metabolic state (*i.e.*, obesity and MetS status). The same trend was observed for fasting insulin levels and SBP. The Ov/Ob subjects with the MetS had a more deteriorated plasma lipid profile including increased plasma TG levels and decreased plasma HDL-C levels compared to Ov/Ob without MetS or NW individuals. There was no significant difference between groups for plasma LDL-C levels. NW individuals had lower HOMA-IR index values than Ov/Ob with or without MetS.

### 3.2. Serum Metabolite Score

**Figure 1:**
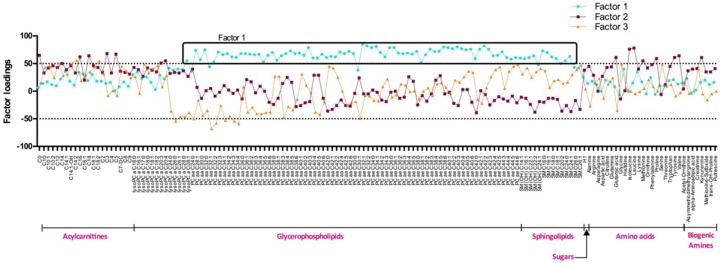
Metabolites patterns of the INFOGENE study. Metabolites with absolute factor loadings ≥0.5 or −0.5 were regarded as significant contributors to the pattern. The blue line represents factor 1, the purple factor 2 and the orange factor 3.

Using PCA, we retained three main factors that explained a maximum of variance in the data (Figure 1). Factor 1 was composed of 81 metabolites, including long chain PCaa and PCae, and few medium to long chain SGs. Factor 2 was composed of short and long chain ACs and several AA (C0, C16, C18, C3, C4, glutamic acid, isoleucine, leucine, methionine, phenylalanine, tyrosine and valine), whereas factor 3 was composed of few medium chain ACs (C12, C14:1-OH, C14:2, C18:2). Lists of metabolites composing each factor are available in Tables S1–S3.

In Table 2, factor scores are presented according to BMI and MetS status (Table 2A), and according to bioelectrical impedance analysis (BIA) measurement and MetS status (Table 2B). In fact, since body composition is difficult to predict based on a single indicator such as BMI, we also classified the subjects as being obese or not according to their percentage of body fat mass assessed by BIA. Briefly, when the BMI status was used, factor 1 score was higher among Ov/Ob participants with MetS (0.36 ± 1.01) than among Ov/Ob participants without MetS (0.01 ± 1.01) or NW participants (−0.31 ± 0.84). Factor 2 score was higher among NW subjects (0.14 ± 1.03) than among Ov/Ob subjects with (−0.22 ± 0.73) or without (−0.05 ± 1.04) MetS. NW subjects had higher factor 3 scores (0.34 ± 0.95) than Ov/Ob subjects with (−0.36 ± 0.95) or without MetS (−0.01 ± 0.99). When the fat percentage was used, factor 1 score was higher among Ov/Ob participants with MetS (0.30 ± 0.16) than among Ov/Ob participants without MetS (0.03 ± 0.11) or NW participants (−0.35 ± 0.14). Factor 2 score was higher among NW subjects (0.29 ± 0.14) than among Ov/Ob subjects with (0.03 ± 0.17) or without (−0.08 ± 0.12) MetS. However, associations with factor 3 were lost. The association of obesity, metabolic syndrome and metabolite profile remained similar in the present study sample even though BMI or BIA measurements are used to define obesity. Thus, for the rest of the study, the obesity will be defined using BMI criteria.

### 3.3. Relation between Cardiometabolic Risk Factors and PCA Factors

Table 3 shows that factor 1 was positively correlated with total-C and TG levels in all three groups. HDL-C was also positively correlated with factor 1 but only in subjects without MetS (Ov/Ob: *r* = 0.27; *p* = 0.01 and NW: *r* = 0.26; *p* = 0.04) while LDL-C was correlated with factor 1 only in Ov/Ob people with MetS (*r* = 0.31; *p* = 0.04). In Table 4, we observe that factor 2 was negatively correlated to WC among Ov/Ob with MetS (*r* = −0.36; *p* = 0.02). Factor 2 was also associated with HDL-C (*r* = 0.34; *p* = 0.008) and insulin levels (*r* = −0.25; *p* = 0.05) in NW individuals without MetS. In addition, factor 2 was associated with LDL-C (*r* = 0.30; *p* = 0.007) and total-C (*r* = 0.34; *p* = 0.002) among Ov/Ob subjects without MetS. Finally, Table 5 shows that factor 3 is negatively associated with glucose among Ov/Ob with MetS (*r* = −0.30; *p* = 0.05) and with insulin (*r* = −0.27; *p* = 0.02) and HOMA-IR (*r* = −0.24; *p* = 0.03) among Ov/Ob without MetS.

## 4. Discussion

Abdominal obesity is a feature of MetS. The risk of developing metabolic disorders is proportional to the degree of obesity [19]. However, all obese individuals do not have MetS [4,20]. Mechanisms explaining the development of MetS are poorly understood and must be intensely investigated since their understanding may favor the design of prevention and therapeutic strategies. Metabolites are the intermediate products of different metabolic pathways. In the present study, employing metabolomics, we investigated differences in plasma composition between lean and Ov/Ob people, with or without MetS.

Obesity is defined as an excess of body fat. Unfortunately, body fat is tricky to measure. Several methods can be used to determine whether a human being is obese or not [21]. Depending on the method used, the definition of obesity can be surprisingly different. In the present study, obesity status was assessed using BMI and fat mass quantification by BIA. Metabolite score remained similar when either BMI or percent body fat were used. Thus, the rest of the analyses were done using BMI criteria to classify study subjects as being obese or not.

Using PCAs, we identified three metabolite clusters: factor 1, 2 and 3. Factor 1 is composed of long chain phosphatidylcholines and SMs. It regroups metabolites that seem to be linked with MetS but also with obesity itself. In fact, factor 1 score was higher and positive among Ov/Ob participants with and without MetS and negative among NW people. PCaa and SMs composing factor 1 are involved in lipid metabolism and are important for lipid transport. Their concentrations have been related to fat accumulation in liver of obese subjects [22], possibly explaining higher plasma levels of these metabolites in this population. In a study realised by Eisingerand *et al*., an increase of total PCs and SMs was observed in mice fed a high fat diet in comparison with mice fed with a standard chow diet [23].

Plasmalogens (PLs) are a subclass of GPs characterized by the presence of a vinyl-ether bond and an ester bond at the sn-1 and sn-2 positions, respectively, of the glycerol backbone [24]. They have several cell functions; in fact, they act as reservoirs for second messengers or as structural attributes in the plasma membrane. They are also anti-oxidants preventing lipoprotein peroxidation; the hydrogen atoms adjacent to the vinyl-ether bond have relatively low disassociation energies and are preferentially oxidized over diacyl GP when exposed to various free radicals and singlet oxygen. PLs are consumed in this reaction. This was proposed to spare the oxidation of polyunsaturated fatty acids and other vulnerable membrane lipids, suggesting a role for PLs as sacrificial oxidants [25]. Sinder and collaborators showed that the oxidative products of PLs are unable to further propagate lipid peroxidation; thus PLs may terminate lipid oxidation. In the present study, a global reduction of PL concentrations was seen among Ov/Ob subjects (Table S1) in line with increased oxidative state observed in obesity [26]. To investigate environmental factors related to obesity, Pietilainen *et al*. led a study in monozygotic twins discordant for obesity. A change in the general metabolites serum composition was seen between obese and non-obese twins [27]. A decrease of ether phospholipids was observed among obese twins compared to lean twins [27,28]. Jordan *et al*. also reported a diminution of PCae with an increase of subject’s fat mass. Studies among children have shown a decrease of PCae between lean and obese children [9,29]. As far as PCaa are concerned, Oberbach *et al*. showed an increase of several PCaa including C32:0, C32:1 and C40:5 levels among obese as compared to lean individuals [30,31], a trend that was also observed for PCaa C32:1 and C40:5 in the present study. Globally, our current study adds to others arguing that development of obesity comes along with a global decrease of PCae and with an increase of several PCaa by demonstrating that these changes are more important in the group of subjects with MetS.

Factor 2 is composed of AA including branch chained AA (BCAAs) (isoleucine, leucine and valine) and few ACs. BCAAs are essential AA that must be supplied by the diet [32]. BCAAs play a variety of physiological roles. They are regulatory molecules that have several cellular functions. They are nutrient signals that regulate protein synthesis, degradation and insulin secretion [33,34,35]. Although BCAAs are essential to the human body, studies have shown a dysregulation of their plasma concentrations with diseases [15]. For instance, in obese and insulin resistant subjects, an increase of BCAA levels is often observed when compared to lean individuals [30,36,37]. Factor 2 was comprised of a few ACs including free and odd short-chain ACs (C3 and C5). These two ACs are by-products of BCAA catabolism, thus their presence may be related to the degradation of BCAAs.

Factor 2 and factor 3 scores are higher among NW than among Ov/Ob individuals. It seems that the clusters of metabolites belonging to factors 2 and 3 are more tightly linked to a healthy metabolic state than factor 1. Several authors reported that increased plasma BCAA and AC levels were linked to several metabolic diseases [32,38]. In the present study, factor 2 and 3 scores were higher among NW subjects with a more favorable metabolic state. Factor 2 and 3 metabolite scores were lower among Ov/Ob participants with MetS and intermediate for Ov/Ob without MetS. This suggests a relation between metabolites composing these factors, MetS and obesity status. In the present study, an increase of the BCAA levels can be seen from NW participants to Ov/Ob ones, this increase being more important among Ov/Ob subjects with MetS. The same situation was observed for short chain ACs such as C0, C3 and C5.

Correlations between factor 1 and cardiometabolic risk factors were investigated. A positive correlation of factor 1 with plasma total-C and TG levels was observed in each group. This suggests that the association between factor 1, total-C and TG is independent of the metabolic state of study participants. A positive correlation was also seen between factor 1 and LDL-C levels but only among Ov/Ob participants with MetS. LDL-C is an established risk factor for CVDs but is not included as one of the five components of the MetS [39]. In literature, evidence regarding the association between LDL-C levels and MetS is not abundant, thus the contribution of LDL-C levels to the altered plasma lipid profile in MetS is unclear. HDL-C levels are known to be negatively associated with a deteriorated metabolic health. Here, we observed a positive association between factor 1 and HDL-C levels but only in groups without MetS irrespective of their obesity status. Factor 1 discriminates participants according to their MetS status rather than on obesity status. Factor 1 is mostly composed of GPs and SGs. Since these lipids are present in membranes and are involved in lipoprotein metabolism and transport, the association between blood lipids and these metabolites was expected. In a study led by Graessen *et al*., bariatric surgery was associated with an improvement of the plasma lipid profile. In fact, they reported an improvement of the LDL-C levels along with a decrease of PC concentrations in obese subjects with MetS [15,40].

Factor 2 is composed of AA including BCAAs and few ACs. BCAAs have several physiological functions [41,42]. However, despite these beneficial effects on metabolic health, studies have shown that increased levels of circulating BCAAs were associated with poor metabolic health. In the present study, factor 2 was positively correlated with HDL-C and negatively correlated with insulin levels among NW participants without MetS. A negative correlation was also noted between WC and factor 2 but only among Ov/Ob with MetS. These results are concordant with what was observed with the metabolite scores; in fact, NW participants had higher metabolite scores and lower concentrations of BCAAs and short chain ACs. The association of factor 2 with a better health profile seems to be positively linked to HDL-C and negatively to insulin levels. Factor 3 is composed of medium ACs and is negatively correlated with glucose levels among Ov/Ob subjects with MetS and also negatively correlated to insulin levels and HOMA-IR among Ov/Ob individuals without MetS. In a study led by our group, a cluster of metabolites composed of medium to long chain ACs was negatively associated to a Western dietary pattern [43]. These results strengthen the hypothesis that the medium to long chain AC parts of factor 3 are positively associated with metabolic health.

## 5. Conclusions

Using metabolomic profiling, we identified a handful of relevant metabolites distinguishing obesity and MetS. For instance, long chain PCaa, PCae and medium chain SMs composing factor 1 were distributed differently between Ov/Ob and the other groups. They seem to be rather associated with a deteriorated metabolic profile. In contrast, factors 2 and 3 are associated with a healthy metabolic profile. So far, mechanisms explaining the shift of metabolite levels from normal to abnormal metabolic phenotypes are not clear. More validation studies are needed to better understand the underlying mechanisms triggering the dysregulation of metabolic traits in obesity.

## Figures and Tables

**Table 1:** Baseline characteristics.

	MetS−		MetS+	*p*-Value
	Ov/Ob (*n* = 83)	NW (*n* = 65)	Ov/Ob (*n* = 46)	
Age (years)	35.7 ± 10.4 ^2^	28.9 ± 7.4 ^1^	37.9 ± 10.0 ^3^	<0.0001
BMI (kg/m^2^)	31.4 ± 4.2 ^2^	22.2 ± 1.8 ^1^	34.3 ± 4.9 ^3^	<0.0001
Waist circumference (cm) ^a^	97.8 ± 10.8 ^2^	74.7 ± 5.7 ^1^	109.0 ± 11.9 ^3^	<0.0001
Fat mass (kg)	31.3 ± 9.0 ^2^	14.5 ± 4.3 ^1^	36.5 ± 10.5 ^3^	<0.0001
Lean mass (kg)	57.5 ± 11.1 ^2^	48.9 ± 8.9 ^1^	65.3 ± 12.1 ^3^	<0.0001
Systolic blood pressure (mmHg)	119.9 ± 9.0 ^2^	115.9 ± 9.8 ^1^	130.1 ± 10.6 ^3^	<0.0001
Diastolic blood pressure (mmHg)	77.7 ± 7.7 ^1^	74.0 ± 9.9 ^1^	83.5 ± 9.5 ^2^	<0.0001
Fasting glucose (mmol/L)	5.4 ± 0.5 ^2^	5.8 ± 1.3 ^1^	6.1 ± 1.1 ^3^	0.002
Fasting insulin (pmol/L)	86.1 ± 56.3 ^2^	48.7 ± 17.1 ^1^	133.1 ± 72.3 ^3^	<0.0001
HOMA-IR	2.98 ± 1.77 ^2^	1.84 ± 0.94 ^1^	5.33 ± 3.70 ^3^	<0.0001
Total-C (mmol/L)	4.57 ± 1.00 ^2^	4.13 ± 0.67 ^1^	4.84 ± 1.14 ^2^	0.02
LDL-C (mmol/L)	2.81 ± 0.91	2.52 ± 0.70	2.99 ± 1.23	0.29
HDL-C (mmol/L)	1.32 ± 0.30 ^2^	1.60 ± 0.45 ^1^	0.99 ± 0.24 ^3^	<0.0001
TG (mmol/L)	1.15 ± 0.56 ^2^	0.77 ± 0.31 ^1^	2.16 ± 1.21 ^3^	<0.0001

Values are means ± SD. ^a^ *p* values are adjusted for age, sex and BMI. *p* values in bold were considered significantly different. ^1,2,3^ Represents the differences between groups using generalized linear models (GLM). Abbreviations: Ov/Ob, overweight/obese; NW, Normal weight; MetS+, with the metabolic syndrome; MetS−, without the metabolic syndrome.

**Table 2A:** Metabolite factor scores (obesity classification of subjects according to BMI criteria).

	MetS−		MetS+	*p*-Value
	Ov/Ob (*n* = 83)	NW (*n* = 65)	Ov/Ob (*n* = 46)	
Factor 1	0.01 ± 1.01 ^2^	−0.31 ± 0.84 ^3^	0.36 ± 1.01 ^1^	**0.003**
Factor 2	−0.05 ± 1.04 ^2^	0.14 ± 1.03 ^3^	−0.22 ± 0.73 ^1^	**0.02**
Factor 3	−0.01 ± 0.99 ^2^	0.34 ± 0.95 ^3^	−0.36 ± 0.95 ^1^	**0.03**

**Table 2B:** Metabolite factor scores (obesity classification of subjects according to BIA criteria).

	MetS−		MetS+	*p*-Value
	Ob (*n* = 84)	NW (*n* = 65)	Ob (*n* = 36)	
Factor 1	0.03 ± 0.11 ^3^	−0.35 ± 0.14 ^2^	0.30 ± 0.16 ^1^	**0.005**
Factor 2	−0.20 ± 0.11 ^1^	0.29 ± 0.14 ^2^	−0.17 ± 0.16 ^1^	**0.03**
Factor 3	−0.08 ± 0.12 ^1^	0.12 ± 0.15 ^1^	0.03 ± 0.17 ^1^	0.59

Values are means ± SD. ^1,2,3^ Represents the differences between groups using the least squares means. *p* value in bold were considered significantly different. Abbreviations: Ov/Ob, overweight/obese; NW, Normal weight; MetS+, with the metabolic syndrome; MetS−, without the metabolic syndrome.

**Table 3:** Correlations between cardiometabolic risk factors and factor 1.

		MetS−		MetS+
		Ov/Ob (*n* = 83)	NW (*n* = 65)	Ov/Ob (*n* = 46)
**WC**	*r*	0.14	0.23	0.01
	*p*	0.23	0.07	0.95
**Total-C**	*r*	0.32	0.38	0.63
	*p*	**0.003**	**0.003**	**<0.0001**
**TG**	*r*	0.61	0.45	0.44
	*p*	**<0.0001**	**0.0002**	**0.003**
**HDL-C**	*r*	0.27	0.26	0.19
	*p*	**0.01**	**0.04**	0.22
**LDL-C**	*r*	0.11	0.22	0.31
	*p*	0.33	0.09	**0.04**
**Glucose**	*r*	−0.03	0.03	0.16
	*p*	0.81	0.81	0.32
**Insulin**	*r*	0.12	−0.05	−0.03
	*p*	0.39	0.69	0.86
**SBP**	*r*	0.03	−0.07	0.23
	*p*	0.81	0.64	0.19
**DBP**	*r*	−0.1	−0.04	0.16
	*p*	0.39	0.74	0.29
**HOMA-IR**	*r*	0.08	−0.03	0.002
	*p*	0.47	0.82	0.91

Pearson correlation coefficients (*r*) and associated *p* values are provided. Abbreviations: Ov/Ob, overweight/obese; NW, Normal weight; MetS+, with the metabolic syndrome; MetS−, without the metabolic syndrome.

**Table 4:** Correlations between cardiometabolic risk factors and factor 2.

		MetS−		MetS+
		Ov/Ob (*n* = 83)	NW (*n* = 65)	Ov/Ob (*n* = 46)
**WC**	*r*	−0.10	0.09	−0.36
	*p*	0.35	0.47	**0.02**
**Total-C**	*r*	0.34	0.18	0.17
	*p*	**0.002**	0.17	0.28
**TG**	*r*	0.01	−0.09	0.05
	*p*	0.91	0.46	0.75
**HDL-C**	*r*	0.09	0.34	0.12
	*p*	0.42	**0.008**	0.53
**LDL-C**	*r*	0.30	−0.02	0.16
	*p*	**0.007**	0.91	0.31
**Glucose**	*r*	−0.02	0.002	0.14
	*p*	0.83	0.99	0.36
**Insulin**	*r*	0.08	−0.25	0.15
	*p*	0.49	**0.05**	0.32
**SBP**	*r*	0.05	−0.04	0.09
	*p*	0.67	0.79	0.58
**DBP**	*r*	0.09	0.08	0.06
	*p*	0.44	0.56	0.71
**HOMA-IR**	*r*	0.07	−0.14	0.16
	*p*	0.54	0.30	0.30

Pearson correlation coefficients (*r*) and associated *p* values are provided. Abbreviations: Ov/Ob, overweight/obese; NW, Normal weight; MetS+, with the metabolic syndrome; MetS−, without the metabolic syndrome.

**Table 5:** Correlations between cardiometabolic risk factors and factor 3.

		MetS−		MetS+
		Ov/Ob (*n* = 83)	NW (*n* = 65)	Ov/Ob (*n* = 46)
**WC**	*r*	−0.07	0.02	0.17
	*p*	0.55	0.89	0.28
**Total-C**	*r*	−0.06	0.14	0.13
	*p*	0.62	0.29	0.41
**TG**	*r*	−0.16	0.04	−0.22
	*p*	0.16	0.75	0.15
**HDL-C**	*r*	0.16	0.22	0.14
	*p*	0.16	0.09	0.36
**LDL-C**	*r*	−0.08	0.01	0.11
	*p*	0.46	0.93	0.48
**Glucose**	*r*	0.0009	−0.10	−0.32
	*p*	0.99	0.42	**0.05**
**Insulin**	*r*	−0.27	−0.21	−0.1
	*p*	**0.02**	0.09	0.52
**SBP**	*r*	0.07	0.06	−0.06
	*p*	0.51	0.67	0.71
**DBP**	*r*	−0.04	0.01	0.25
	*p*	0.71	0.92	0.11
**HOMA-IR**	*r*	−0.24	−0.23	−0.17
	*p*	**0.03**	0.08	0.26

Pearson correlation coefficients (*r*) and associated *p* values are provided. Abbreviations: Ov/Ob, overweight/obese; NW, Normal weight; MetS+, with the metabolic syndrome; MetS−, without the metabolic syndrome.

## Supplementary Materials

The following are available online at http://www.mdpi.com/2072-6643/8/6/324/s1, Table S1: Factor 1 metabolites composition categorized by obesity and MetS status, Table S2: Factor 2 metabolites composition categorized by obesity and MetS status, Table S3: Factor 3 metabolites composition categorized by obesity and MetS status.
