# Supplementary material for: Association between Metabolite Profiles, Metabolic Syndrome and Obesity Status
Source: Nutrients. 2016 May 27;8(6):324. doi: 10.3390/nu8060324 (PMC4924165; doi:10.3390/nu8060324)
Supplement: Supplementary file 1 [file nutrients-08-00324-s001.docx]

Supplementary Materials: Association between Metabolite Profiles, Metabolic Syndrome and Obesity Status

Bénédicte Allam-Ndoul, Frédéric Guénard, Véronique Garneau, Hubert Cormier, Olivier Barbier, Louis Pérusse and Marie-Claude Vohl

**Table S1.** Factor 1 metabolites composition categorized by obesity and MetS status.

|  | **MetS−** | | **MetS+** | ***p* Value** |
| --- | --- | --- | --- | --- |
|  | **Ov/Ob (*n* = 83)** | **NW (*n* = 65)** | **Ov/Ob (*n* = 46)** |  |
| **Metabolites** | |  |  |  |
| **Acylcarnitines** | |  |  |  |
| **C10:1** | 0.15 *±* 0.005 | 0.15 *±* 0.004 | 0.14 *±* 0.006 | 0.42 |
| **C10:2** | 0.03 *±* 0.0007 | 0.03 *±* 0.0008 | 0.03 *±* 0.001 | 0.52 |
| **C14** | 0.04 *±* 0.001 | 0.04 *±* 0.001 | 0.04 *±* 0.001 | 0.73 |
| **C14:1** | 0.11 *±* 0.004 | 0.12 *±* 0.004 | 0.11 *±* 0.005 | 0.7 |
| **lyso Pca** |  |  |  |  |
| **C16:0** | 72.69 *±* 1.96 | 79.05 *±* 2.32 | 71.94 *±* 2.70 | 0.07 |
| **Pcaa** |  |  |  |  |
| **C32:0** | 14.33 *±* 0.36 | 14.23 *±* 0.42 | 14.74 *±* 0.50 | 0.72 |
| **C32:1** | 20.68 *±* 1.12 ^1^ | 17.71 *±* 1.32 ^1^ | 24.95 *±* 1.54 ^2^ | **0.0031** |
| **C32:3** | 0.7 *±* 0.02 | 0.67 *±* 0.02 | 0.69 *±* 0.03 | 0.26 |
| **C34:1** | 233.43 *±* 6.68 ^1^ | 223.91 *±* 7.89 ^2^ | 246.68 *±* 9.2 ^3^ | **0.003** |
| **C34:2** | 461.02 *±* 10.56 ^1^ | 450.22 *±* 12.48 ^2^ | 478 *±* 14.54 ^3^ | **0.009** |
| **C34:3** | 22.92 *±* 0.76 ^1^ | 21.09 *±* 0.90 ^1^ | 24.4 *±* 1.04 ^2^ | **0.016** |
| **C34:4** | 2.38 *±* 0.09 ^1^ | 1.98 *±* 0.11 ^2^ | 2.51 *±* 0.13 ^1^ | **0.005** |
| **C36:0** | 2.4 *±* 0.08 ^1^ | 2.54 *±* 0.09 ^1^ | 2.04 *±* 0.11 ^2^ | **0.0035** |
| **C36:1** | 49.89 *±* 1.24 | 48.5 *±* 1.46 | 52.69 *±* 1.70 | 0.19 |
| **C36:2** | 261.9 *±* 5.67 | 256.54 *±* 6.70 | 271.8 *±* 7.81 | 0.35 |
| **C36:3** | 172.96 *±* 4.57 | 170.22 *±* 5.40 | 178.89 *±* 6.30 | 0.59 |
| **C36:4** | 219.17 *±* 6.44 ^1^ | 201.29 *±* 7.61 ^2^ | 231.92 *±* 8.86 ^3^ | **0.04** |
| **C38:0** | 3.23 *±* 0.10 ^2^ | 3.64 *±* 0.12 ^1^ | 2.82 *±* 0.14 ^1^ | **0.0002** |
| **C38:1** | 0.88 *±* 0.03 ^2^ | 1.09 *±* 0.04 ^1^ | 0.79 *±* 0.05 ^2^ | **<0.0001** |
| **C38:3** | 48.18 *±* 1.31 ^2^ | 42.19 *±* 1.55 ^1^ | 51.75 *±* 1.80 ^3^ | **0.0005** |
| **C38:4** | 100.48 *±* 2.62 ^2^ | 89.66 *±* 3.10 ^1^ | 107.45 *±* 3.61 ^2^ | **0.0014** |
| **C38:5** | 69.35 *±* 1.90 | 65.92 *±* 2.24 | 70.94 *±* 2.61 | 0.34 |
| **C38:6** | 91.8 *±* 3.20 | 95.73 *±* 3.78 | 86.88 *±* 4.40 | 0.34 |
| **C40:1** | 0.37 *±* 0.009 ^2^ | 0.41 *±* 0.01 ^1^ | 0.33 *±* 0.01 ^3^ | **<0.0001** |
| **C40:3** | 0.56 *±* 0.013 ^1^ | 0.57 *±* 0.015 ^1^ | 0.51 *±* 0.018 ^2^ | **0.03** |
| **C40:4** | 3.43 *±* 0.10 ^1^ | 3.22 *±* 0.12 ^1^ | 3.78 *±* 0.14 ^2^ | **0.016** |
| **C40:5** | 9.62 *±* 0.29 ^1^ | 9.04 *±* 0.34 ^1^ | 10.45  *±* 0.40 ^2^ | **0.04** |
| **C40:6** | 26.1 *±* 0.87 | 25.93 *±* 1.03 | 26.33 *±* 1.20 | 0.97 |
| **C42:0** | 0.58 *±* 0.02 ^2^ | 0.68 *±* 0.02 ^1^ | 0.5 *±* 0.03 ^3^ | **<0.0001** |
| **C42:1** | 0.28 *±* 0.01 ^2^ | 0.33 *±* 0.01 ^1^ | 0.24 *±* 0.01 ^3^ | **<0.0001** |
| **C42:4** | 0.18 *±* 0.004 | 0.18 *±* 0.005 | 0.17 *±* 0.006 | 0.47 |
|  | **Ov/Ob (*n* = 83)** | **NW (*n* = 65)** | **Ov/Ob (*n* = 46)** |  |
| **C42:5** | 0.36 *±* 0.01 | 0.38 *±* 0.01 | 0.35 *±* 0.15 | 0.43 |
| **C42:6** | 0.41 *±* 0.01 ^1^ | 0.43 *±* 0.01 ^1^ | 0.38 *±* 0.01 ^2^ | **0.045** |
| **Pcae** |  |  |  |  |
| **C30:0** | 0.42 *±* 0.01 | 0.43 *±* 0.01 | 0.39 *±* 0.01 | 0.18 |
| **C30:1** | 0.19 *±* 0.01 | 0.17 *±* 0.02 | 0.19 *±* 0.02 | 0.72 |
| **C32:1** | 2.85 *±* 0.07 | 2.8 *±* 0.08 | 2.68 *±* 0.09 | 0.34 |
| **C32:2** | 0.8 *±* 0.02 ^1^ | 0.8 *±* 0.03 | 0.73 *±* 0.03 ^2^ | 0.1 |
| **C34:0** | 1.55 *±* 0.04 | 1.58 *±* 0.05 | 1.47 *±* 0.05 | 0.31 |
| **C34:1** | 10.78 *±* 0.24 | 10.96 *±* 0.3 | 10.44 *±* 0.03 | 0.52 |
| **C34:2** | 12.93 *±* 0.33 ^2^ | 13.16 *±* 0.40 ^1^ | 11.67 *±* 0.46 ^3^ | **0.04** |
| **C34:3** | 8.6 *±* 0.27 ^2^ | 9.59 *±* 0.31 ^1^ | 7.23 *±* 0.37 ^3^ | **<0.0001** |
| **C36:0** | 0.94 *±* 0.02 ^2^ | 0.93 *±* 0.03 | 0.86 *±* 0.03 ^3^ | 0.14 |
| **C36:1** | 8.27 *±* 0.20 | 8.6 *±* 0.23 | 7.86 *±* 0.27 | 0.12 |
| **C36:2** | 16.2 *±* 0.40 ^1^ | 17.96 *±* 0.48 ^1^ | 15.16 *±* 0.56 ^2^ | **0.016** |
| **C36:3** | 8.85 *±* 0.23 ^1^ | 9.29 *±* 0.28 ^1^ | 8.07 *±* 0.32 ^2^ | **0.02** |
| **C36:4** | 19.92 *±* 0.54 | 18.19 *±* 0.64 | 18.92 *±* 0.75 | 0.12 |
| **C36:5** | 13.6 *±* 0.40 | 12.57 *±* 0.48 | 12.65 *±* 0.56 | 0.24 |
| **C38:0** | 3.06 *±* 0.10 | 3.17 *±* 0.12 | 2.84 *±* 0.14 | 0.11 |
| **C38:1** | 0.62 *±* 0.2 | 0.67 *±* 0.03 | 0.59 *±* 0.03 | 0.78 |
| **C38:3** | 4.75 *±* 0.12 | 4.78 *±* 0.14 | 4.63 *±* 0.17 | 0.6 |
| **C38:4** | 14.57 *±* 0.36 | 14.35 *±* 0.42 | 13.95 *±* 0.50 | 0.23 |
| **C38:5** | 21.24 *±* 0.50 | 20.74 *±* 0.60 | 20.23 *±* 0.70 | 0.49 |
| **C38:6** | 9.27 *±* 0.26 | 9.28 *±* 0.30 | 8.38 *±* 0.35 | 0.09 |
| **C40:1** | 1.45 *±* 0.04 ^1^ | 1.56 *±* 0.05 ^1^ | 1.37 *±* 0.06 ^2^ | **0.05** |
| **C40:2** | 1.67 *±* 0.03 ^1^ | 1.68 *±* 0.04 ^1^ | 1.42 *±* 0.05 ^2^ | **0.0005** |
| **C40:3** | 1.1 *±* 0.02 ^1^ | 1.15 *±* 0.03 ^1^ | 0.99 *±* 0.03 ^2^ | **0.0016** |
| **C40:4** | 2.43 *±* 0.05 ^1^ | 2.51 *±* 0.06 ^1^ | 2.24 *±* 0.7 ^2^ | **0.03** |
| **C40:5** | 3.51 *±* 0.08 ^2^ | 3.9 *±* 0.1 ^1^ | 3.23 *±* 0.11 ^3^ | **0.0001** |
| **C40:6** | 4.59 *±* 0.12 ^2^ | 5.27 *±* 0.14 ^1^ | 4.13 *±* 0.17 ^3^ | **<0.0001** |
| **C40:2** | 0.68 *±* 0.01 ^1^ | 0.72 *±* 0.02 ^1^ | 0.65 *±* 0.02 ^3^ | **0.05** |
| **C42:1** | 0.35 *±* 0.01 | 0.35 *±* 0.01 | 0.33 *±* 0.01 | 0.39 |
| **C42:2** | 0.54 *±* 0.01 ^1^ | 0.56 *±* 0.01 ^1^ | 0.5^2^ *±* 0.02 | 0.11 |
| **C42:3** | 0.79 *±* 0.02 ^2^ | 0.87 *±* 0.02 ^1^ | 0.65 *±* 0.03 ^3^ | **<0.001** |
| **C42:4** | 0.95 *±* 0.02 ^2^ | 1.02 *±* 0.03 ^1^ | 0.84 *±* 0.03 ^3^ | **0.0007** |
| **C42:5** | 2.31 *±* 0.05 ^2^ | 2.5 *±* 0.07 ^1^ | 2.1 *±* 0.08 ^3^ | **0.003** |
| **C44:3** | 0.09 *±* 0.002 ^1^ | 0.1 *±* 0.003 ^2^ | 0.09 *±* 0.003 ^1^ | 0.016 |
|  | **MetS−** | | **MetS+** | ***p* Value** |
|  | **Ov/Ob (*n* = 83)** | **NW (*n* = 65)** | **Ov/Ob (*n* = 46)** |  |
| **C44:4** | 0.37 *±* 0.02 ^2^ | 0.4 *±* 0.01 ^1^ | 0.34 *±* 0.01 ^2^ | **0.008** |
| **C44:5** | 1.78 *±* 0.05 ^1^ | 1.87 *±* 0.06 ^1^ | 1.61 *±* 0.07 ^3^ | **0.04** |
| **C44:6** | 1.25 *±* 0.04 ^2^ | 1.38 *±* 0.04 ^1^ | 1.13 *±* 0.05 ^3^ | **0.005** |
| **SM(OH)** |  |  |  |  |
| **C16:1** | 3.64 *±* 0.08 ^1^ | 3.69 *±* 0.10 ^1^ | 3.23 *±* 0.1 ^2^ | **0.0082** |
| **C22:1** | 14.76 *±* 0.33 ^2^ | 15.51 *±* 0.40 ^1^ | 13.72 *±* 0.46 ^3^ | **0.02** |
| **C22:2** | 12.42 *±* 0.28 ^2^ | 13.23 *±* 0.32 ^1^ | 10.95 *±* 0.38 ^3^ | **0.0001** |
| **C24:1** | 1.19 *±* 0.03 ^1^ | 1.25 *±* 0.03 ^1^ | 1.01 *±* 0.04 ^2^ | **0.0006** |
| **SM** |  |  |  |  |
| **C16:0** | 121.44 *±* 2.04 ^1^ | 123.35 *±* 2.42 ^1^ | 109.73 *±* 2.82 ^2^ | **0.0008** |
| **C16:1** | 19.56 *±* 0.35 ^2^ | 17.94 *±* 0.42 ^1^ | 18.46 *±* 0.49 ^2^ | **0.01** |
| **C18:0** | 23.4 *±* 0.51 | 21.87 *±* 0.60 | 22.35 *±* 0.71 | 0.14 |
| **C18:1** | 12.05 *±* 0.28 | 11.19 *±* 0.33 | 11.56 *±* 0.39 | 0.14 |
| **C24:0** | 20.23 *±* 0.47 ^1^ | 21.03 *±* 0.56 ^1^ | 18.87 *±* 0.65 ^2^ | **0.05** |
| **C24:1** | 50.52 *±* 1.06 ^1^ | 50.82 *±* 1.26 ^1^ | 45.28 *±* 1.47 ^2^ | **0.008** |

Values are means *±* SD. Abbreviations: Ov/Ob, overweight/obese; NW, Normal weight; MetS+, with the metabolic syndrome; MetS−, without the metabolic syndrome. *p* value in bold were considered significantly different. ^1,2,3^ Represents the differences between groups using GLM models.

**Table S2.** Factor 2 metabolites composition categorized by obesity and MetS status.

|  | **MetS−** | | **MetS+** | ***p* Value** |
| --- | --- | --- | --- | --- |
| **Facteur 2** | **Ov/Ob (*n* = 83)** | **NW (*n* = 65)** | **Ov/Ob (*n* = 46)** | |
| **Metabolites** | |  |  |  |
| **Acylcarnitines** | |  |  |  |
| C0 | 35.05 *±* 0.82 ^1^ | 33.06 *±* 0.97 ^1^ | 37.22 *±* 1.12 ^2^ | **0.03** |
| C3 | 0.33 *±* 0.01 ^2^ | 0.29 *±* 0.01 ^1^ | 0.37 *±*  0.01 ^3^ | **0.0004** |
| C:16 | 0.09 *±* 0.003 | 0.08 *±* 0.003 | 0.09 *±* 0.002 | 0.23 |
| C:18 | 0.03 *±* 0.0009 | 0.03 *±* 0.013 | 0.03 *±* 0.0011 | 0.44 |
| **LysoPC** |  |  |  |  |
| C20:3 | 1.70 *±* 0.06 | 1.78 *±* 0.07 | 1.8 *±* 0.08 | 0.59 |
| C20:4 | 5.08 *±* 0.17 | 5.24 *±* 0.20 | 5.06 *±* 0.24 | 0.82 |
| **Amino acids** | |  |  |  |
| leucine | 149.47 *±* 2.90 ^2^ | 140.83 *±* 3.42 ^1^ | 159.62 *±* 3.99 ^3^ | **0.0031** |
| isoleucine | 74.00 *±* 1.47 ^2^ | 68.87 *±* 1.73 ^1^ | 81.04 *±* 2.01 ^3^ | **<0.0001** |
| proline | 187.61 *±* 5.92 | 174.45 *±* 6.99 | 193.52 *±* 8.15 | 0.19 |
| tyrosine | 68.02 *±* 1.27 | 64.74 *±* 1.12 | 70.42 *±* 1.45 | 0.21 |
| methionine | 27.23 *±* 0.58 | 27.61 *±* 0.69 | 27.22 *±* 0.80 | 0.9 |
| valine | 239.95 *±* 5.00 ^2^ | 207.07 *±* 5.92 ^1^ | 258.23 *±* 6.90 ^3^ | **<0.0001** |

Abbreviations: Ov/Ob, overweight/obese; NW, Normal weight; MetS+, with the metabolic syndrome; MetS−, without the metabolic syndrome. *p* value in bold were considered significantly different. ^1,2,3^ Represents the differences between groups using GLM models.

**Table S3.** Factor 3 metabolites composition categorized by obesity and MetS status.

|  | **MetS−** | | **MetS+** | ***p* Value** |
| --- | --- | --- | --- | --- |
| **Facteur 3** | **Ov/Ob (*n* = 83)** | **NW (*n* = 65)** | **Ov/Ob (*n* = 46)** |  |
| **Acylcarnitines** | |  |  |  |
| C12 | 0.1 *±* 0.004 | 0.1 *±* 0.005 | 0.09 *±* 0.004 | 0.11 |
| C14:1-OH | 0.015 *±* 0.0004 ^1^ | 0.016 *±* 0.0005 ^1^ | 0.014 *±* 0.006 ^2^ | **0.03** |
| C14:2 | 0.03 *±* 0.0014 | 0.03 *±* 0.0017 | 0.03 *±* 0.002 | 0.44 |
| C18:2 | 0.33 *±* 0.001 | 0.29 *±* 0.001 | 0.37 *±* 0.001 | 0.5 |

Abbreviations: Ov/Ob, overweight/obese; NW, Normal weight; MetS+, with the metabolic syndrome; MetS−, without the metabolic syndrome. *P* value in bold were considered significantly different. ^1,2^ Represents the differences between groups using GLM models.
